# Synthesis, characterization, and crystal structure of 2-(2-azido­phen­yl)-3-oxo-3*H*-indole 1-oxide

**DOI:** 10.1107/S2056989024001440

**Published:** 2024-02-20

**Authors:** Pawan Dhote, Srinu Tothadi, Chepuri V. Ramana

**Affiliations:** a Organic Chemistry Division, CSIR-National Chemical Laboratory, Pune, 411008, India; b Analytical and Environmental Sciences Division and Centralized Instrumentation Facility, CSIR-Central Salt and Marine Chemicals Research Institute, Gijubhai Badheka Marg, Bhavnagar, 364002 , India; Indian Institute of Science Education and Research Bhopal, India

**Keywords:** crystal structure, isatogen, reactivity, hydrogen bonding

## Abstract

An attempt to explore the reactivity of the nitro group in the presence of gold catalysis in comparison to the azide group yielded intriguing results. Surprisingly, only the nitro group exhibited reactivity, ultimately giving rise to the formation of the title isatogen, C_14_H_8_N_4_O_2_. In the crystal structure, weak C—H⋯O hydrogen bonds and π–π stacking inter­actions link the mol­ecules. The structure exhibits disorder of the mol­ecule.

## Abstract 2

An attempt to explore the reactivity of the nitro group in the presence of gold catalysis in comparison to the azide group yielded intriguing results. Surprisingly, only the nitro group exhibited reactivity, ultimately giving rise to the formation of the title isatogen.

## 1. Chemical context

2,2-Disubstituted indolin-3-ones play a crucial role as fundamental structural motifs in various natural alkaloids and bioactive mol­ecules (Dhote, Patel *et al.*, 2021 ▸; Ji *et al.*, 2019 ▸; Gu *et al.*, 2014 ▸). Thus, substantial research efforts have been dedicated to the synthesis of these essential compounds (Dhote, Patel *et al.*, 2021 ▸; Wang *et al.*, 2021 ▸; Liu & McWhorter, 2003 ▸; Wetzel & Gagosz, 2011 ▸; Liu *et al.*, 2003 ▸). These synthetic methods can be broadly sorted into three main strategies, *viz*. oxidative dearomatization of indoles (Wang *et al.*, 2021 ▸; Liu *et al.*, 2020 ▸), cyclization reactions (Dhote, Pund & Ramana, 2021 ▸; Xie *et al.*, 2021 ▸; Fu & Song, 2018 ▸) and nucleophilic additions to 3*H*-indol-3-ones or indolone-*N*-oxides (Zhang *et al.*, 2017 ▸; Liu *et al.*, 2003 ▸; Berti *et al.*, 1975 ▸). Notably, indolone-*N*-oxides, also known as isatogens, hold substantial importance in medicinal chemistry and serve as inter­mediates in the synthesis of natural alkaloids and bioactive compounds (Nepveu *et al.*, 2010 ▸). The literature contains a wide array of techniques for synthesizing isatogens, encompassing both metal-free and metal-catalyzed routes (Dhote & Ramana, 2021 ▸; Dhote, Halnor *et al.*, 2021 ▸). These methods have been rigorously explored and well documented, underscoring the adaptability and importance of isatogens in the realms of organic synthesis and medicinal chemistry. Over the past few years, our research group has been deeply involved in this field, particularly focusing on their synthesis through nitro­alkyne cyclo­isomerization (Dhote, Pund & Ramana, 2021 ▸; Dhote & Ramana, 2019 ▸; Kumar & Ramana, 2014 ▸, 2015 ▸; Patel *et al.*, 2010 ▸) and we have demonstrated their utility in total synthesis endeavors (Patel *et al.*, 2014 ▸; Reddy & Ramana, 2013 ▸; Kumar *et al.*, 2012 ▸; Patel & Ramana, 2012 ▸).

As part of our efforts to demonstrate the reactivity of the nitro group compared to the azide group (Dhote & Ramana, 2022 ▸; Dhote, Halner *et al.*, 2021 ▸), we designed a substrate that incorporates both a nitro group and an azide group positioned *ortho* to an alkyne functionality. Inter­estingly, when we subjected this substrate to treatment with either Au^III^ or Au^I^, we obtained isatogen **2** with the azide moiety intact in relatively good yield (see reaction scheme[Chem scheme1] below). The structural characterization of 2-(2-azido­phen­yl)-3-oxo-3*H*-indole 1-oxide, **2**, was achieved through spectral and analytical data analysis. In the ^1^H NMR spectra of **2**, the protons were observed to be overlapping, posing challenges in confirming the precise structure. Subsequently, the ^13^C NMR spectrum indicated the absence of the alkyne carbon signal, suggesting a modification at the alkyne functionality (see Figs. S1 and S2 in the supporting information). Additionally, signals corresponding to the carbonyl and the newly formed quaternary center were observed at *δ* = 185.2 and 140.2 ppm, respectively. The mol­ecular composition of compound **2** was further verified as C_14_H_9_N_4_O_2_ through high-resolution mass spectrometry ([*M* + H]^+^ found as 265.0771). Moreover, the structure of **2** was conclusively confirmed through single-crystal X-ray diffraction analysis. 
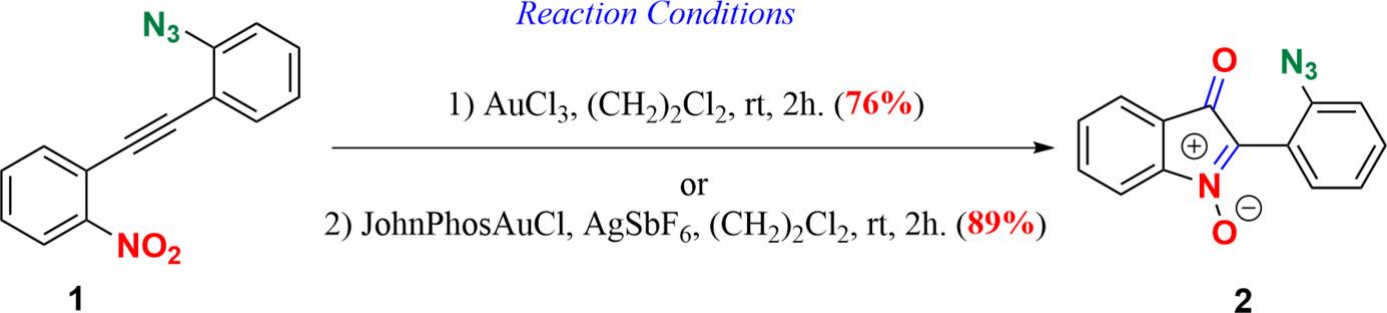


## 2. Structural commentary and supra­molecular features

**Figure 1:**
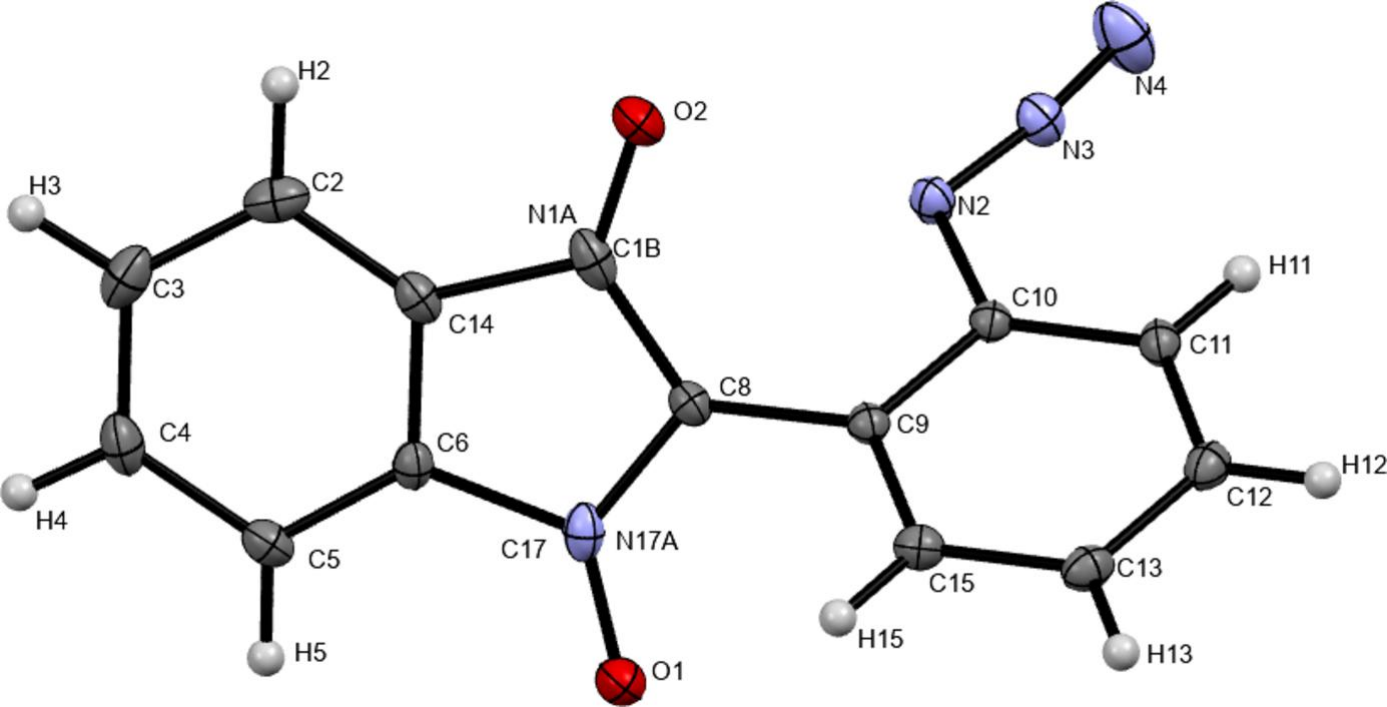
*ORTEP* diagram of **2** with 50% probability displacement ellipsoids. Only one mol­ecule is present in the asymmetric unit.

**Figure 2:**
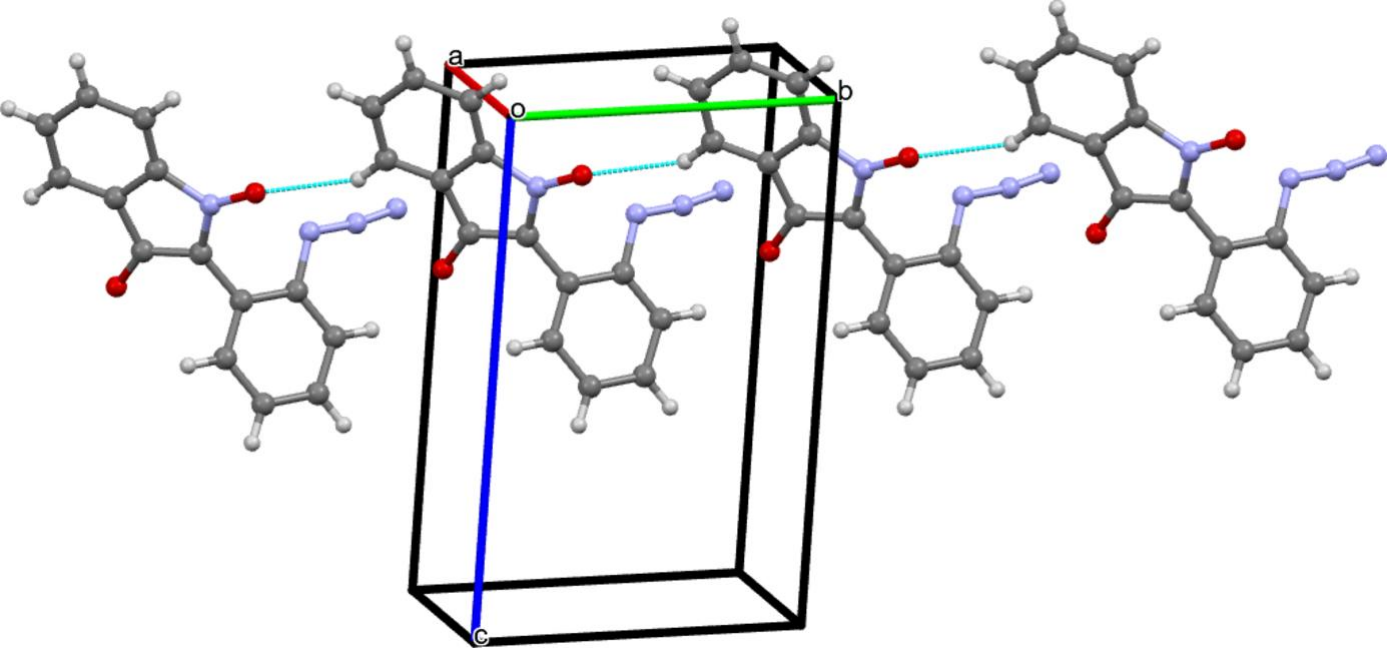
The packing of mol­ecules *via* C—H⋯O hydrogen bonds along the *b*-axis direction. The blue lines depict the inter­molecular inter­actions.

**Figure 3:**
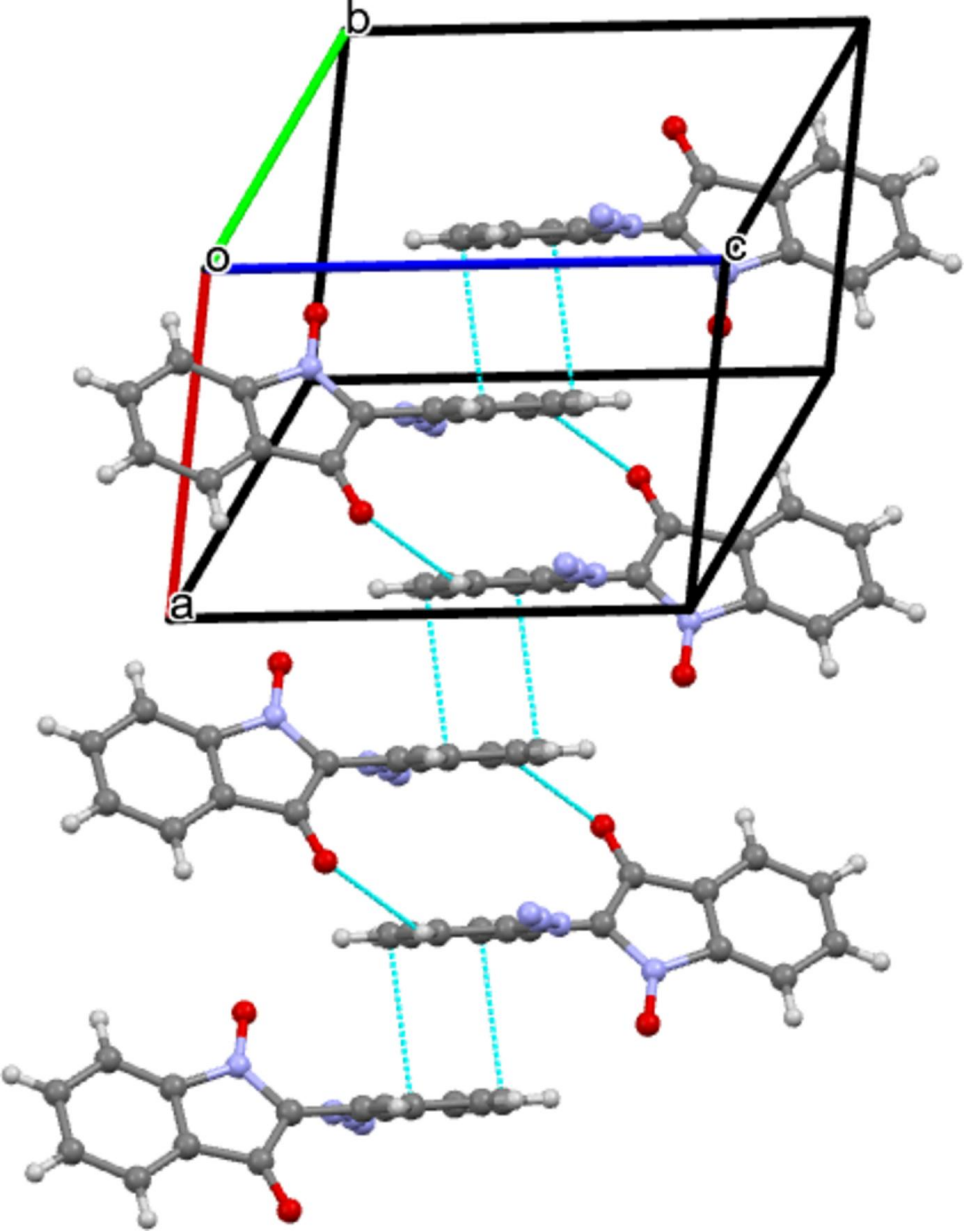
The single-crystal structure of **2**. π–π stacking [centroid–centroid distance = 3.354 (2) Å] can be seen along the *a*-axis direction.

The title compound crystallizes in space group *P*-1 with one mol­ecule in the asymmetric unit (Fig. 1 ▸). Mol­ecules are further connected *via* C—H⋯O (Table 1 ▸) weak hydrogen bonds (Desiraju *et al.*, 1999 ▸) along the *b*-axis direction (Fig. 2 ▸). Moreover, π–π stacking [3.354 (2) Å; Chipot *et al.*, 1996 ▸; Chen *et al.*, 2018 ▸) is observed along the *a*-axis direction (Fig. 3 ▸). However, there are no classical hydrogen bonds present in the crystal packing.

In general, C=O bond lengths (1.22 Å) are always shorter than N^+^—O^−^ (1.26 Å). However, in the current scenario, the C=O and N^+^—O^−^ bond lengths are almost equal (Table 2 ▸). We therefore analyzed the bond lengths in similar structures found in the Cambridge Structural Database (Conquest 2023.3.0; CSD version 5.45, update of November 2023; Groom *et al.*, 2016 ▸), among which seven show N/C disorder and two have similar bond lengths to those in the title compound. We analyzed the bond lengths of similar mol­ecules in the CSD database and further modeled the disorder in the current mol­ecule. After modeling the disorder, the *R* factor reduced to 4.22%. Predominantly, if the atoms of mol­ecules are disordered, the bond distance are averaged out and shows the mean distance of bonds. The N (N1*A* and N17*A*) and C (C1*B* and C17) atoms of isatogen are disordered over two positions with equal (0.5) site occupancy. As the mol­ecule exhibits disorder, the bond distances were averaged out, giving N17*A*—O1/C17=O1 = 1.252 (1) Å and C1*B*=O2/N1*A*—O2 = 1.248 (1) Å.

## 3. Hirshfeld surface analysis

**Figure 4:**
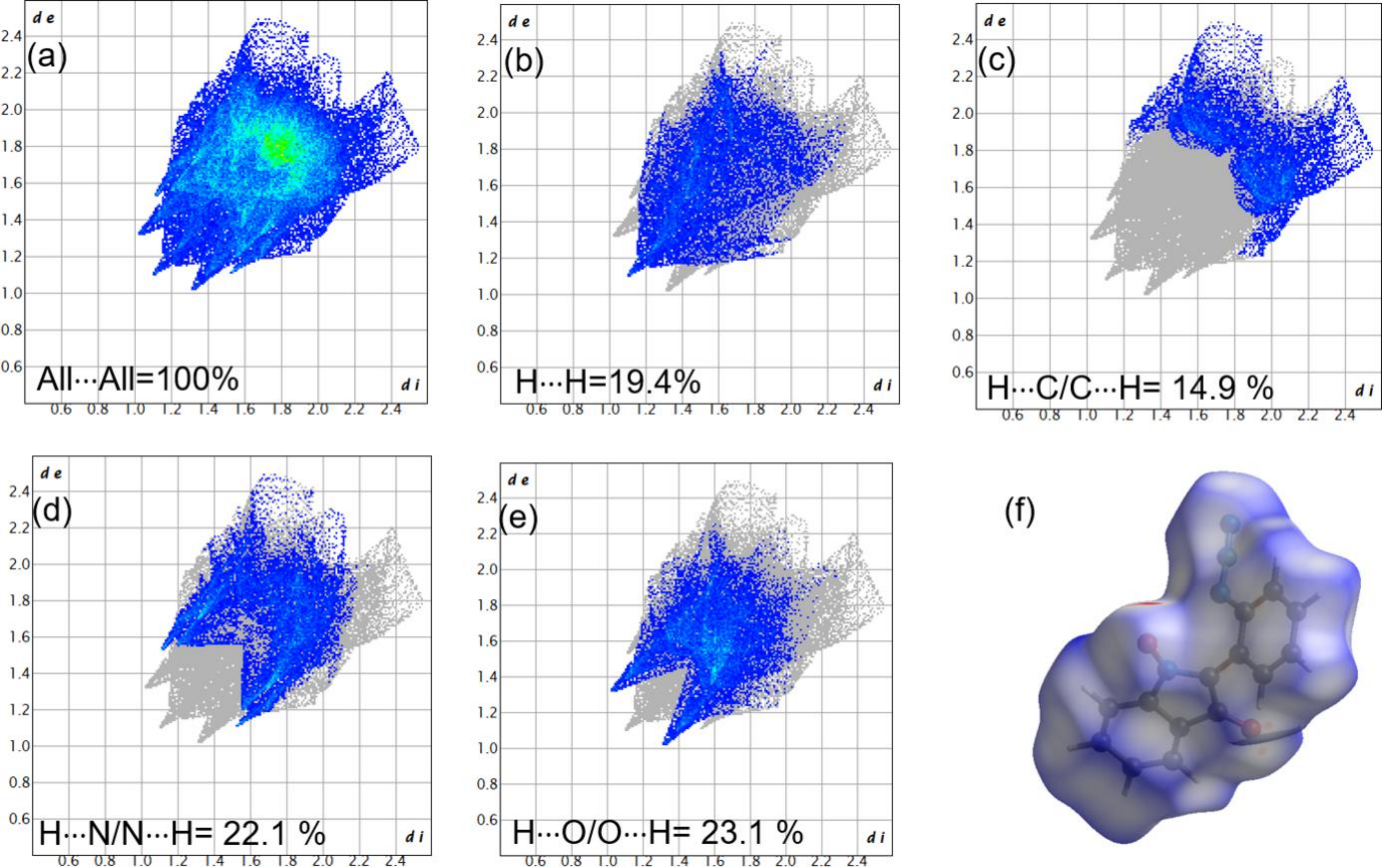
The two-dimensional fingerprint plots for **2**, showing inter­actions: (*a*) all inter­actions and delineated into (*b*) H⋯H contacts; (*c*) H⋯C/C⋯H contacts; (*d*) H⋯N/N⋯H contacts and (*e*) H⋯O/O⋯H contacts; (*f*) Hirshfeld surface mapped with *d*_norm_.

A Hirshfeld surface analysis of compound **2** was undertaken with *CrystalExplorer 21.5* (Spackman *et al.*, 2021 ▸) to investigate the inter­molecular inter­actions. The overall 2D fingerprint plot is shown in Fig. 4 ▸ *a* and those delineated into H⋯H (19.4%), H⋯C/C⋯H (14.9%), H⋯N/N⋯H (22.1%), and H⋯O/O⋯H (23.1%) inter­actions are shown in Fig. 4 ▸ *b*–*e*. Inter­actions such as N⋯O/O⋯N O⋯C/C⋯O and N⋯C/ C⋯N contribute very little to the overall surface and hence those contacts are not shown. The Hirshfeld surface mapped with *d*_norm_ is shown in Fig. 4 ▸ *f* (*d*_norm_ is the normalized sum of *d*_e_ and *d*_i_ where *d_e_* is the distance from the Hirshfeld surface to the nearest atom *i* inter­nal to the surface and *d*_i_ is distance from Hirshfeld surface to the nearest atom *e* external to the surface).

## 4. Database survey

There are twenty structures of isatogen present in the Cambridge Structural Database (CSD; Conquest 2023.3.0; CSD version 5.45, update of November 2023; Groom *et al.*, 2016 ▸), among which ten show N/C disorder. Bond lengths associated with these atoms are unusual. In most crystal structures, C=O is always less than N^+^—O^−^. In contrast, the C=O and N^+^—O^−^ bond lengths in SAWYAR (Clegg, 2005 ▸) and SAZQIU (Clegg & Elsegood, 2005 ▸) are almost equal and are similar to those the title compound (Table 2 ▸).

## 5. Synthesis and crystallization

The reaction was carried out at room temperature and under an argon atmosphere. To a solution of the active gold complex prepared from JohnPhosAuCl (5 mol%) and AgSbF_6_ (10 mol%) or AuCl_3_ (5 mol%) in 1,2-DCE (1 ml) was added a solution of 1-azido-2-[(2 nitro­phen­yl)ethyn­yl]benzene, **1**, in 1,2-DCE (0.5 ml) dropwise over 5 minutes. The resulting solution was stirred for a period of 2 h and then concentrated under reduced pressure. The resulting crude product was purified by column chromatography to afford 2-(2-azido­phen­yl)-3-oxo-3*H*-indole 1-oxide, **2**, as a yellow solid. Next, single crystals were grown by slow evaporation of a solution of compounds (10 mg) in aceto­nitrile (1 ml) [placed in a long glass vial of 2 ml volume and closed with a cotton plug] at room temperature in a dark place.

## 6. Refinement

Crystal data, data collection and structure refinement details are summarized in Table 3 ▸. H atoms were positioned geometrically (C—H = 0.95 Å) and refined as riding with *U*_iso_(H) = 1.2*U*_eq_(C).

## Supplementary Material

Crystal structure: contains datablock(s) I. DOI: 10.1107/S2056989024001440/dx2056sup1.cif

Structure factors: contains datablock(s) I. DOI: 10.1107/S2056989024001440/dx2056Isup2.hkl

Supporting information. DOI: 10.1107/S2056989024001440/dx2056sup4.docx

Supporting information file. DOI: 10.1107/S2056989024001440/dx2056Isup4.cml

CCDC reference: 2303503

Additional supporting information:  crystallographic information; 3D view; checkCIF report

## Figures and Tables

**Table 1:** Hydrogen-bond geometry (Å, °)

*D*—H⋯*A*	*D*—H	H⋯*A*	*D*⋯*A*	*D*—H⋯*A*
C5—H5⋯O2^i^	0.95	2.43	3.1077 (16)	128
C11—H11⋯O1^ii^	0.95	2.57	3.2327 (17)	127

Symmetry codes: (i) 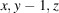 ; (ii) 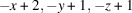 .

**Table 2:** Covalent C=O/N^+^—O^−^ bond lengths (Å) in **2** and related structures

Compound	C=O/N^+^—O^−^	N^+^—O^−^/C=O
SAWYAR	1.240 (4)	1.241 (4)
SAZQIU	1.253 (2)	1.243 (4)
**2**	1.252 (1)	1.248 (1)

**Table 3:** Experimental details

Crystal data	
Chemical formula	C_14_H_8_N_4_O_2_
*M*_r_	264.24
Crystal system, space group	Triclinic, *P* 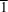
Temperature (K)	100
*a*, *b*, *c* (Å)	7.166 (2), 7.686 (3), 12.172 (4)
α, β, γ (°)	95.473 (16), 105.226 (12), 113.116 (13)
*V* (Å^3^)	579.9 (3)
*Z*	2
Radiation type	Mo *K*α
μ (mm^−1^)	0.11
Crystal size (mm)	0.12 × 0.06 × 0.05

Data collection	
Diffractometer	Bruker D8 VENTURE Kappa Duo PHOTON II CPAD
Absorption correction	Multi-scan (*SADABS*; Krause *et al.*, 2015 ▸)
*T*_min_, *T*_max_	0.626, 0.745
No. of measured, independent and observed [*I* > 2σ(*I*)] reflections	45548, 4818, 3946
*R*_int_	0.049
(sin θ/λ)_max_ (Å^−1^)	0.811

Refinement	
*R*[*F* ^2^ > 2σ(*F* ^2^)], *wR*(*F* ^2^), *S*	0.042, 0.122, 1.05
No. of reflections	4818
No. of parameters	181
H-atom treatment	H-atom parameters constrained
Δρ_max_, Δρ_min_ (e Å^−3^)	0.46, −0.29

Computer programs: *APEX2* and *SAINT* (Bruker, 2015 ▸), *SHELXT2014/5* (Sheldrick, 2015*a* ▸), *SHELXL* (Sheldrick, 2015*b* ▸) and *OLEX2* (Dolomanov *et al.*, 2009 ▸).

## supplementary crystallographic information

### Crystal data

**Table:**

C_14_H_8_N_4_O_2_	*Z* = 2
*M**_r_* = 264.24	*F*(000) = 272
Triclinic, *P*1	*D*_x_ = 1.513 Mg m^−^^3^
*a* = 7.166 (2) Å	Mo *K*α radiation, λ = 0.71073 Å
*b* = 7.686 (3) Å	Cell parameters from 9975 reflections
*c* = 12.172 (4) Å	θ = 3.1–34.6°
α = 95.473 (16)°	µ = 0.11 mm^−^^1^
β = 105.226 (12)°	*T* = 100 K
γ = 113.116 (13)°	Block, colourless
*V* = 579.9 (3) Å^3^	0.12 × 0.06 × 0.05 mm

### Data collection

**Table:**

Bruker D8 VENTURE Kappa Duo PHOTON II CPAD diffractometer	3946 reflections with *I* > 2σ(*I*)
φ and ω scans	*R*_int_ = 0.049
Absorption correction: multi-scan (SADABS; Krause *et al.*, 2015)	θ_max_ = 35.2°, θ_min_ = 3.0°
*T*_min_ = 0.626, *T*_max_ = 0.745	*h* = −11→11
45548 measured reflections	*k* = −12→12
4818 independent reflections	*l* = −19→19

### Refinement

**Table:**

Refinement on *F*^2^	Primary atom site location: structure-invariant direct methods
Least-squares matrix: full	Hydrogen site location: inferred from neighbouring sites
*R*[*F*^2^ > 2σ(*F*^2^)] = 0.042	H-atom parameters constrained
*wR*(*F*^2^) = 0.122	*w* = 1/[σ^2^(*F*_o_^2^) + (0.066*P*)^2^ + 0.1514*P*] where *P* = (*F*_o_^2^ + 2*F*_c_^2^)/3
*S* = 1.05	(Δ/σ)_max_ < 0.001
4818 reflections	Δρ_max_ = 0.46 e Å^−^^3^
181 parameters	Δρ_min_ = −0.29 e Å^−^^3^
0 restraints	

### Special details

**Table:**

Geometry. All esds (except the esd in the dihedral angle between two l.s. planes) are estimated using the full covariance matrix. The cell esds are taken into account individually in the estimation of esds in distances, angles and torsion angles; correlations between esds in cell parameters are only used when they are defined by crystal symmetry. An approximate (isotropic) treatment of cell esds is used for estimating esds involving l.s. planes.

### Fractional atomic coordinates and isotropic or equivalent isotropic displacement parameters (Å^2^)

**Table:**

	*x*	*y*	*z*	*U*_iso_*/*U*_eq_	Occ. (<1)
O1	0.67828 (11)	−0.03978 (9)	0.35537 (6)	0.01840 (14)	
O2	0.33615 (11)	0.29764 (9)	0.15423 (6)	0.01970 (14)	
N4	1.05131 (16)	0.87935 (13)	0.27516 (9)	0.02787 (19)	
N2	0.81071 (12)	0.56952 (10)	0.28504 (6)	0.01489 (14)	
N3	0.93852 (12)	0.73575 (11)	0.28689 (7)	0.01669 (14)	
C10	0.77636 (12)	0.53396 (11)	0.39246 (7)	0.01172 (14)	
C9	0.64747 (12)	0.34085 (11)	0.39218 (7)	0.01194 (14)	
C8	0.54989 (12)	0.18621 (11)	0.28650 (7)	0.01240 (14)	
N1A	0.40687 (13)	0.17681 (11)	0.17988 (8)	0.01765 (16)	0.5
C14	0.34033 (13)	−0.00926 (12)	0.09961 (7)	0.01469 (15)	
C2	0.20365 (14)	−0.08622 (14)	−0.01379 (8)	0.01915 (17)	
H2	0.134768	−0.017372	−0.055149	0.023*	
C3	0.17122 (15)	−0.27158 (15)	−0.06522 (8)	0.02109 (18)	
H3	0.077902	−0.330058	−0.143374	0.025*	
C11	0.86772 (13)	0.67870 (12)	0.49428 (7)	0.01393 (15)	
H11	0.955317	0.808707	0.493893	0.017*	
C12	0.83019 (14)	0.63213 (13)	0.59624 (7)	0.01570 (15)	
H12	0.891796	0.730900	0.665580	0.019*	
C13	0.70271 (14)	0.44142 (13)	0.59774 (7)	0.01594 (15)	
H13	0.677285	0.410403	0.667753	0.019*	
C15	0.61299 (13)	0.29688 (12)	0.49620 (7)	0.01451 (15)	
H15	0.527337	0.166823	0.497471	0.017*	
C17	0.57040 (12)	0.01171 (11)	0.27811 (7)	0.01585 (15)	0.5
C6	0.44015 (13)	−0.11001 (12)	0.16023 (7)	0.01356 (14)	
C5	0.41071 (14)	−0.29159 (12)	0.11154 (8)	0.01623 (15)	
H5	0.479954	−0.359268	0.153933	0.019*	
C4	0.27263 (15)	−0.37139 (13)	−0.00430 (8)	0.01966 (17)	
H4	0.247984	−0.496292	−0.041850	0.024*	
C1B	0.40687 (13)	0.17681 (11)	0.17988 (8)	0.01765 (16)	0.5
N17A	0.57040 (12)	0.01171 (11)	0.27811 (7)	0.01585 (15)	0.5

### Atomic displacement parameters (Å^2^)

**Table:**

	*U*^11^	*U*^22^	*U*^33^	*U*^12^	*U*^13^	*U*^23^
O1	0.0195 (3)	0.0149 (3)	0.0197 (3)	0.0079 (2)	0.0042 (2)	0.0036 (2)
O2	0.0234 (3)	0.0138 (3)	0.0220 (3)	0.0095 (2)	0.0055 (2)	0.0039 (2)
N4	0.0328 (5)	0.0178 (4)	0.0343 (5)	0.0059 (3)	0.0205 (4)	0.0074 (3)
N2	0.0166 (3)	0.0121 (3)	0.0142 (3)	0.0040 (2)	0.0059 (2)	0.0031 (2)
N3	0.0184 (3)	0.0153 (3)	0.0178 (3)	0.0072 (3)	0.0084 (3)	0.0037 (2)
C10	0.0113 (3)	0.0120 (3)	0.0122 (3)	0.0057 (2)	0.0036 (2)	0.0023 (2)
C9	0.0112 (3)	0.0116 (3)	0.0128 (3)	0.0053 (3)	0.0033 (2)	0.0023 (2)
C8	0.0124 (3)	0.0099 (3)	0.0145 (3)	0.0043 (2)	0.0048 (3)	0.0024 (2)
N1A	0.0196 (4)	0.0103 (3)	0.0231 (4)	0.0038 (3)	0.0121 (3)	0.0015 (3)
C14	0.0162 (3)	0.0110 (3)	0.0183 (4)	0.0053 (3)	0.0088 (3)	0.0031 (3)
C2	0.0170 (4)	0.0261 (4)	0.0194 (4)	0.0118 (3)	0.0078 (3)	0.0116 (3)
C3	0.0172 (4)	0.0269 (4)	0.0129 (3)	0.0052 (3)	0.0033 (3)	0.0012 (3)
C11	0.0134 (3)	0.0120 (3)	0.0145 (3)	0.0053 (3)	0.0028 (3)	0.0004 (3)
C12	0.0165 (3)	0.0178 (4)	0.0130 (3)	0.0096 (3)	0.0028 (3)	0.0008 (3)
C13	0.0175 (4)	0.0202 (4)	0.0130 (3)	0.0104 (3)	0.0056 (3)	0.0044 (3)
C15	0.0148 (3)	0.0152 (3)	0.0153 (3)	0.0074 (3)	0.0057 (3)	0.0050 (3)
C17	0.0135 (3)	0.0138 (3)	0.0155 (3)	0.0015 (3)	0.0058 (3)	−0.0005 (2)
C6	0.0134 (3)	0.0117 (3)	0.0128 (3)	0.0029 (3)	0.0046 (3)	0.0010 (2)
C5	0.0187 (4)	0.0132 (3)	0.0191 (4)	0.0078 (3)	0.0077 (3)	0.0056 (3)
C4	0.0223 (4)	0.0133 (3)	0.0198 (4)	0.0041 (3)	0.0087 (3)	−0.0014 (3)
C1B	0.0196 (4)	0.0103 (3)	0.0231 (4)	0.0038 (3)	0.0121 (3)	0.0015 (3)
N17A	0.0135 (3)	0.0138 (3)	0.0155 (3)	0.0015 (3)	0.0058 (3)	−0.0005 (2)

### Geometric parameters (Å, º)

**Table:**

O1—C17	1.2523 (11)	C14—C1B	1.4767 (12)
O1—N17A	1.2523 (11)	C2—H2	0.9500
O2—N1A	1.2485 (11)	C2—C3	1.4033 (15)
O2—C1B	1.2485 (11)	C3—H3	0.9500
N4—N3	1.1323 (12)	C3—C4	1.3892 (14)
N2—N3	1.2396 (11)	C11—H11	0.9500
N2—C10	1.4245 (11)	C11—C12	1.3876 (12)
C10—C9	1.4061 (12)	C12—H12	0.9500
C10—C11	1.3933 (12)	C12—C13	1.3950 (13)
C9—C8	1.4628 (12)	C13—H13	0.9500
C9—C15	1.3996 (12)	C13—C15	1.3911 (12)
C8—N1A	1.4019 (13)	C15—H15	0.9500
C8—C17	1.4028 (12)	C17—C6	1.4697 (12)
C8—C1B	1.4019 (13)	C6—C5	1.3744 (12)
C8—N17A	1.4028 (12)	C6—N17A	1.4697 (12)
N1A—C14	1.4767 (12)	C5—H5	0.9500
C14—C2	1.3762 (13)	C5—C4	1.4033 (13)
C14—C6	1.3865 (12)	C4—H4	0.9500
			
N3—N2—C10	116.77 (7)	C12—C11—H11	120.1
N4—N3—N2	171.89 (9)	C11—C12—H12	119.7
C9—C10—N2	116.31 (7)	C11—C12—C13	120.52 (8)
C11—C10—N2	123.03 (8)	C13—C12—H12	119.7
C11—C10—C9	120.64 (8)	C12—C13—H13	120.2
C10—C9—C8	121.71 (7)	C15—C13—C12	119.67 (8)
C15—C9—C10	118.70 (7)	C15—C13—H13	120.2
C15—C9—C8	119.59 (7)	C9—C15—H15	119.6
N1A—C8—C9	126.97 (7)	C13—C15—C9	120.76 (8)
N1A—C8—C17	107.79 (7)	C13—C15—H15	119.6
C17—C8—C9	125.04 (7)	O1—C17—C8	127.91 (8)
C1B—C8—C9	126.97 (7)	O1—C17—C6	123.29 (8)
C1B—C8—N17A	107.79 (7)	C8—C17—C6	108.80 (7)
N17A—C8—C9	125.04 (7)	C14—C6—C17	107.50 (7)
O2—N1A—C8	127.81 (8)	C14—C6—N17A	107.50 (7)
O2—N1A—C14	123.50 (8)	C5—C6—C14	122.61 (8)
C8—N1A—C14	108.64 (7)	C5—C6—C17	129.89 (8)
C2—C14—N1A	131.01 (8)	C5—C6—N17A	129.89 (8)
C2—C14—C6	121.74 (8)	C6—C5—H5	121.9
C2—C14—C1B	131.01 (8)	C6—C5—C4	116.25 (8)
C6—C14—N1A	107.23 (8)	C4—C5—H5	121.9
C6—C14—C1B	107.23 (8)	C3—C4—C5	121.38 (8)
C14—C2—H2	121.7	C3—C4—H4	119.3
C14—C2—C3	116.59 (8)	C5—C4—H4	119.3
C3—C2—H2	121.7	O2—C1B—C8	127.81 (8)
C2—C3—H3	119.3	O2—C1B—C14	123.50 (8)
C4—C3—C2	121.42 (8)	C8—C1B—C14	108.64 (7)
C4—C3—H3	119.3	O1—N17A—C8	127.91 (8)
C10—C11—H11	120.1	O1—N17A—C6	123.29 (8)
C12—C11—C10	119.71 (8)	C8—N17A—C6	108.80 (7)
			
O1—C17—C6—C14	178.78 (8)	C14—C6—C5—C4	0.01 (12)
O1—C17—C6—C5	−0.58 (14)	C14—C6—N17A—O1	178.78 (8)
O2—N1A—C14—C2	2.51 (14)	C14—C6—N17A—C8	−0.88 (9)
O2—N1A—C14—C6	−176.07 (8)	C2—C14—C6—C17	−179.07 (7)
N2—C10—C9—C8	−1.48 (11)	C2—C14—C6—C5	0.35 (13)
N2—C10—C9—C15	178.39 (7)	C2—C14—C6—N17A	−179.07 (7)
N2—C10—C11—C12	−178.73 (7)	C2—C14—C1B—O2	2.51 (14)
N3—N2—C10—C9	−174.08 (7)	C2—C14—C1B—C8	−179.99 (9)
N3—N2—C10—C11	4.34 (12)	C2—C3—C4—C5	0.43 (14)
C10—C9—C8—N1A	−59.12 (12)	C11—C10—C9—C8	−179.94 (7)
C10—C9—C8—C17	126.65 (9)	C11—C10—C9—C15	−0.08 (11)
C10—C9—C8—C1B	−59.12 (12)	C11—C12—C13—C15	0.14 (13)
C10—C9—C8—N17A	126.65 (9)	C12—C13—C15—C9	−0.61 (12)
C10—C9—C15—C13	0.57 (12)	C15—C9—C8—N1A	121.02 (9)
C10—C11—C12—C13	0.35 (12)	C15—C9—C8—C17	−53.22 (11)
C9—C10—C11—C12	−0.38 (12)	C15—C9—C8—C1B	121.02 (9)
C9—C8—N1A—O2	0.35 (14)	C15—C9—C8—N17A	−53.22 (11)
C9—C8—N1A—C14	−177.02 (7)	C17—C8—N1A—O2	175.39 (8)
C9—C8—C17—O1	−2.70 (13)	C17—C8—N1A—C14	−1.97 (9)
C9—C8—C17—C6	176.94 (7)	C17—C6—C5—C4	179.29 (8)
C9—C8—C1B—O2	0.35 (14)	C6—C14—C2—C3	−0.31 (13)
C9—C8—C1B—C14	−177.02 (7)	C6—C14—C1B—O2	−176.07 (8)
C9—C8—N17A—O1	−2.70 (13)	C6—C14—C1B—C8	1.43 (9)
C9—C8—N17A—C6	176.94 (7)	C6—C5—C4—C3	−0.39 (13)
C8—C9—C15—C13	−179.56 (7)	C5—C6—N17A—O1	−0.58 (14)
C8—N1A—C14—C2	−179.99 (9)	C5—C6—N17A—C8	179.76 (8)
C8—N1A—C14—C6	1.43 (9)	C1B—C8—N17A—O1	−177.87 (8)
C8—C17—C6—C14	−0.88 (9)	C1B—C8—N17A—C6	1.78 (9)
C8—C17—C6—C5	179.76 (8)	C1B—C14—C2—C3	−178.72 (8)
N1A—C8—C17—O1	−177.87 (8)	C1B—C14—C6—C5	179.09 (7)
N1A—C8—C17—C6	1.78 (9)	C1B—C14—C6—N17A	−0.32 (9)
N1A—C14—C2—C3	−178.72 (8)	N17A—C8—C1B—O2	175.39 (8)
N1A—C14—C6—C17	−0.32 (9)	N17A—C8—C1B—C14	−1.97 (9)
N1A—C14—C6—C5	179.09 (7)	N17A—C6—C5—C4	179.29 (8)
C14—C2—C3—C4	−0.07 (13)		

### Hydrogen-bond geometry (Å, º)

**Table:**

*D*—H···*A*	*D*—H	H···*A*	*D*···*A*	*D*—H···*A*
C5—H5···O2^i^	0.95	2.43	3.1077 (16)	128
C11—H11···O1^ii^	0.95	2.57	3.2327 (17)	127

Symmetry codes: (i) *x*, *y*−1, *z*; (ii) −*x*+2, −*y*+1, −*z*+1.

### Table 2. Weak hydrogen bond table of compound 2

No classical hydrogen bonds found. For C—H···acceptor interactions, see: Steiner, Th., (1996), Cryst. Rev., 6, 1-57 H-Bond classification [Jeffrey, G. A., Maluszynska, H. & Mitra, J., (1985), Int J. Biol. Macromol. 7, 336-348]

**Table:**

S.No	D-H···A	H···A (Å)	D···A (Å)	〈D-H···.A (°)	
1	C5-H5···O2^(a)^	2.43	3.1077 (16)	128	
2	C11-H11···O1^(b)^	2.57	3.2327 (17)	127	

Symmetry codes: (i) x,-1+y,z (ii) 2-x,1-y,1-z .
