# Supplementary material for: Synthesis, characterization, and crystal structure of 2-(2-azido­phen­yl)-3-oxo-3H-indole 1-oxide
Source: Acta Crystallogr E Crystallogr Commun. 2024 Feb 20;80(Pt 3):310–3. doi: 10.1107/S2056989024001440 (PMC10915669; doi:10.1107/S2056989024001440)
Supplement: Supplementary file 3 [file e-80-00310-sup4.docx]

**Synthesis, characterization, and crystal structure of 2-(2-azidophenyl)-3-**

**oxo-3H-indole 1-oxide**

Pawan Dhote,a Srinu Tothadi^b^* and Chepuri V. Ramana^a^*

^a^Organic Chemistry Division, CSIR-National Chemical Laboratory, Pune, 411008, India, and

^b^Analytical and Environmental Sciences Division and

Centralized Instrumentation, Facility, CSIR-Central Salt and Marine Chemicals Research Institute, Gijubhai, Badheka, Marg, Bhavnagar, 364002, India.

Correspondence email: [srinut@csmcri.res.in](mailto:srinut@csmcri.res.in), vr.chepuri@ncl.res.in

**
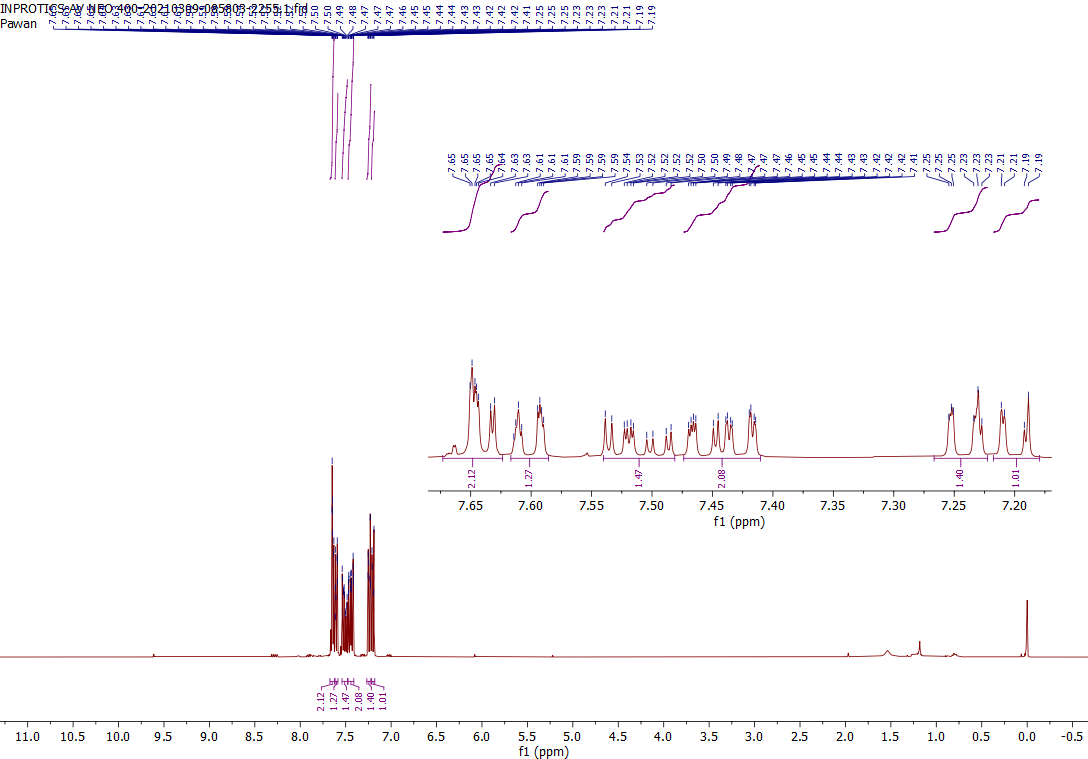
**

**Figure S1**

^1^H NMR (400 MHz, CDCl_3_): d 7.18–7.22 (m, 1H), 7.24 (dt, J = 8.8, 0.8 Hz, 1H), 7.40–7.48 (m, 2H), 7.48–7.55 (m, 1H), 7.59–7.62 (m, 1H), 7.62–7.68 (m, 2H) ppm.


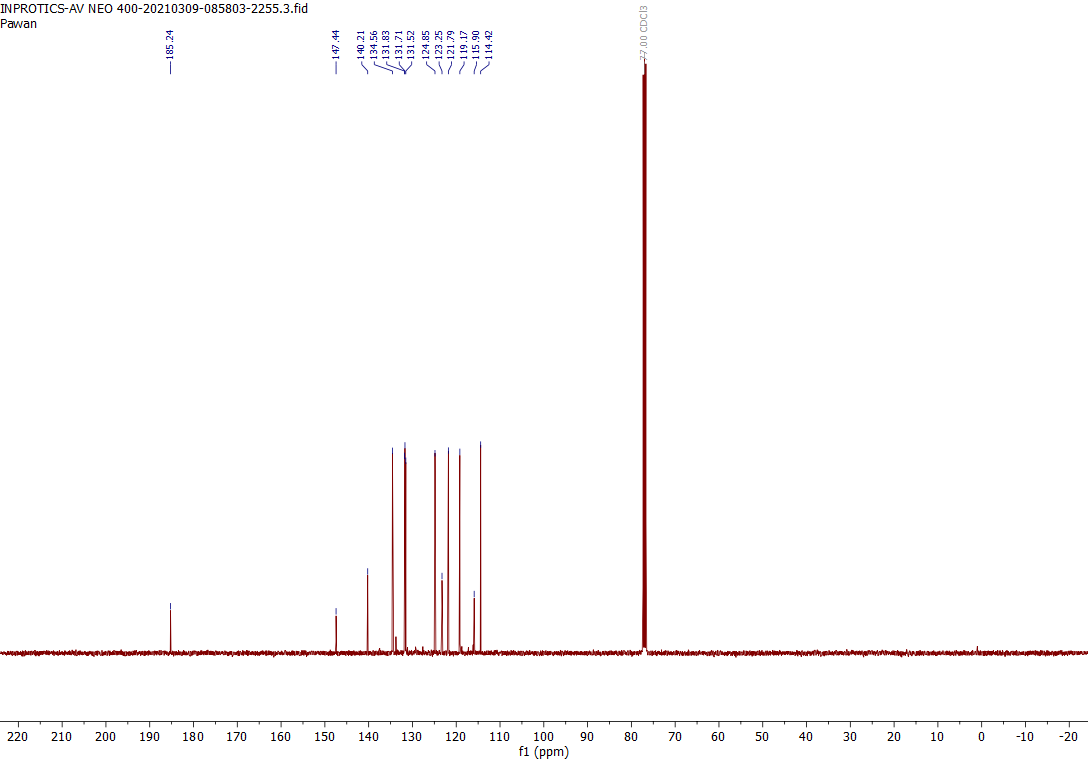


**Figure S2**

^13^C NMR (100 MHz, CDCl_3_): d 114.4 (d), 115.9 (s), 119.2 (d), 121.8 (d), 123.2 (s), 124.8 (d), 131.5 (d), 131.7 (d), 131.8 (d), 134.6 (d), 140.2 (s), 147.4 (s), 185.2 (s) ppm.
